# Supplementary material for: Transcriptomic comparison of early onset preeclampsia and placenta accreta identifies inverse trophoblast and decidua functions at the maternal-fetal interface
Source: NPJ Womens Health. 2025 Oct 24;3(1):59. doi: 10.1038/s44294-025-00106-7 (PMC12549338; doi:10.1038/s44294-025-00106-7)
Supplement: Supplementary file 1 — Supplementary information [file 44294_2025_106_MOESM1_ESM.pdf]

Supplementary information

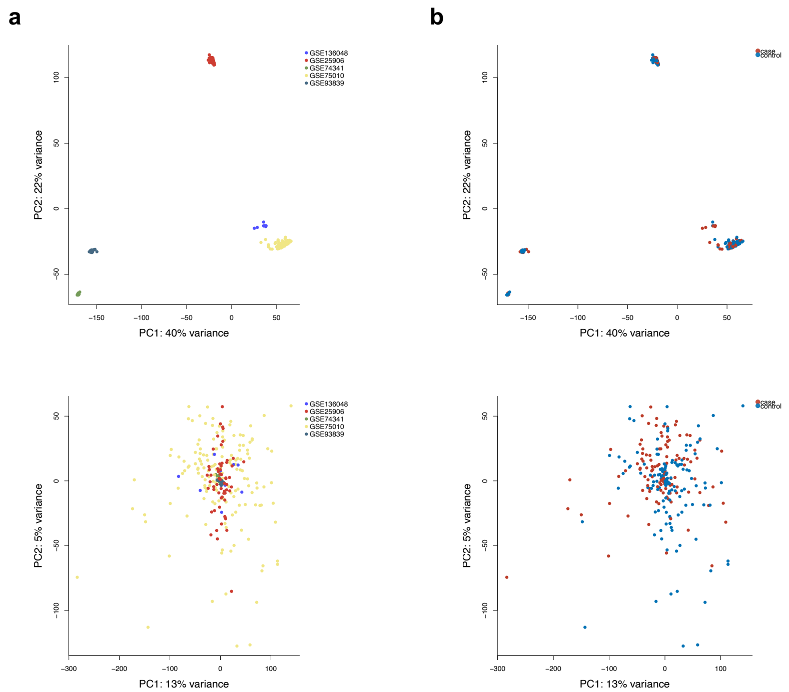

Supplementary Figure 1: Harmonization of five microarray datasets.
a Merging, with study labeled.
b Merging, with case and control labeled.

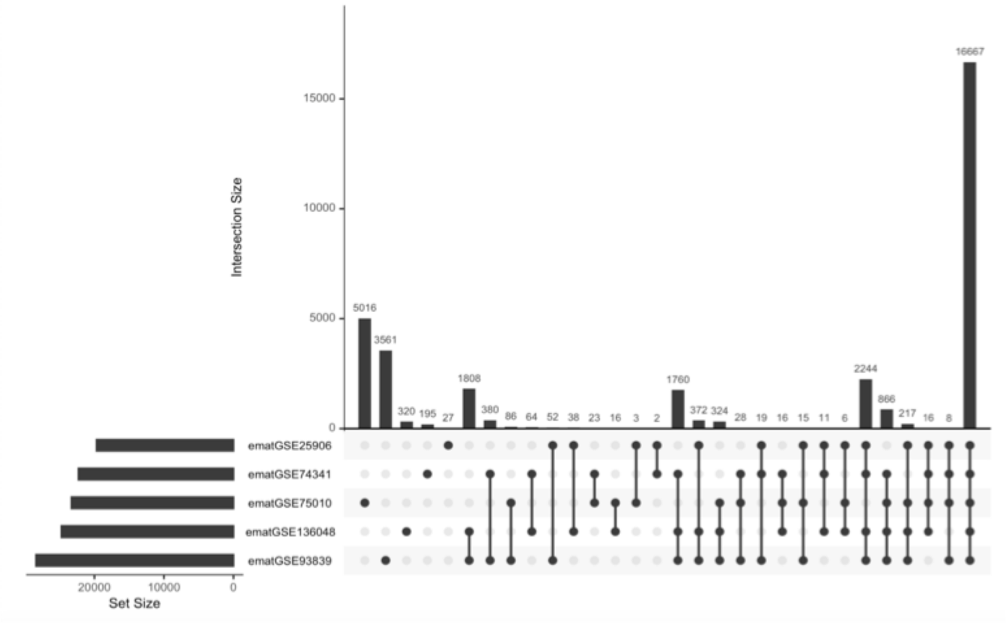

Supplementary Figure 2: Gene overlap in merged dataset.
Upset plot demonstrating the 5 microarray datasets and the overlap in gene set size for each one, resulting in a total of 16,667 shared genes across the 5 microarrays.

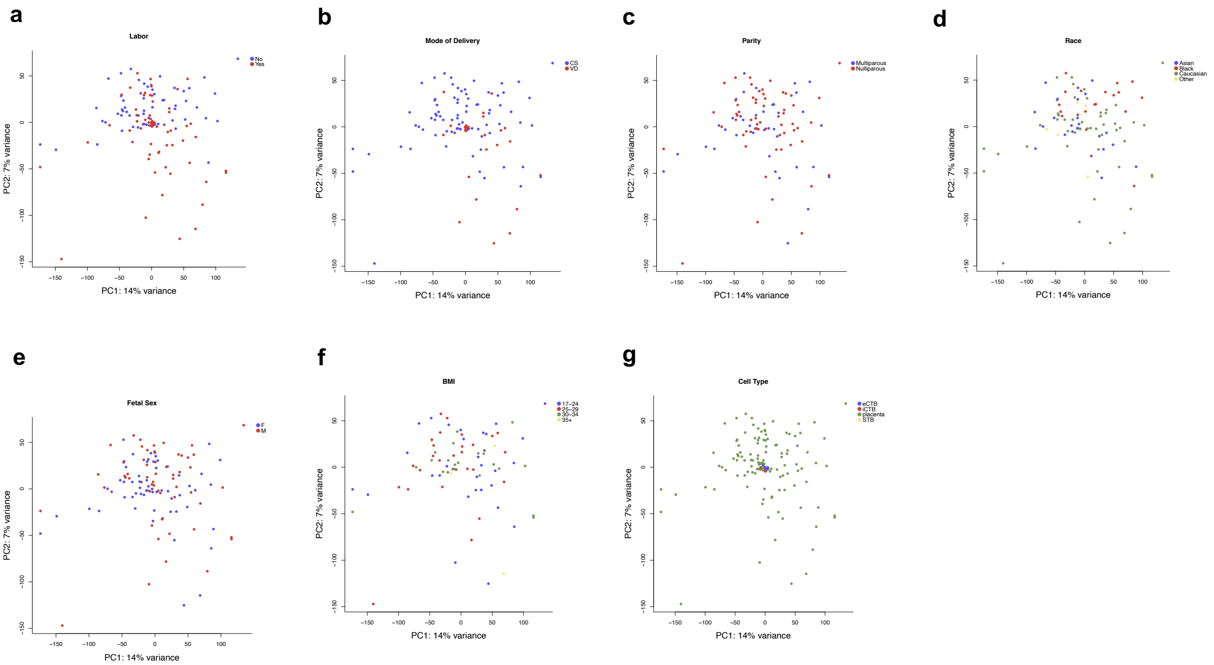

Supplementary Figure 3: PCA plots to explore variance of covariates in merged dataset.

a Labor, p>0.05.

b Mode of delivery, p=0.02. CS: cesarean section. VD: vaginal delivery.

c Parity, p>0.05.

d Race, p>0.05.

e Fetal sex, p>0.05. F: female. M: male.

f Body mass index (BMI, in kg/m^2), p>0.05.

g Cell type, p<0.01. eCTB: endovascular cytotrophoblast. iCTB: invasive cytotrophoblast. STB: syncytiotrophoblast.

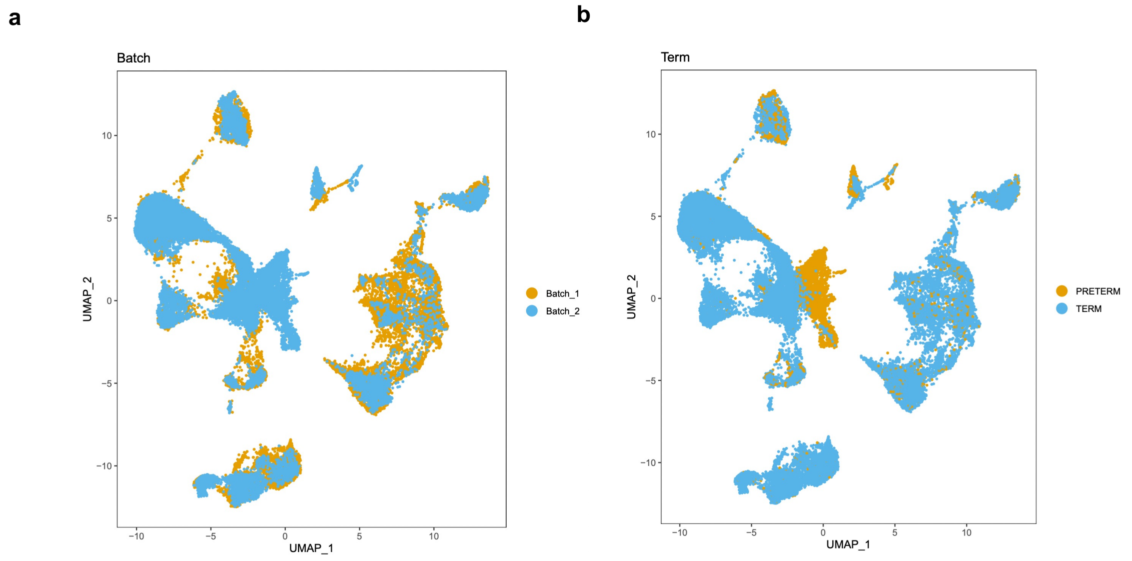

Supplementary Figure 4: Covariate correction for placenta accreta single-cell data.
Placenta accreta single-cell UMAP data with mapping of individual cells by covariate. Both covariates were adjusted for in the final differential gene expression analysis for the single-cell data.
a batch.
b preterm or term.

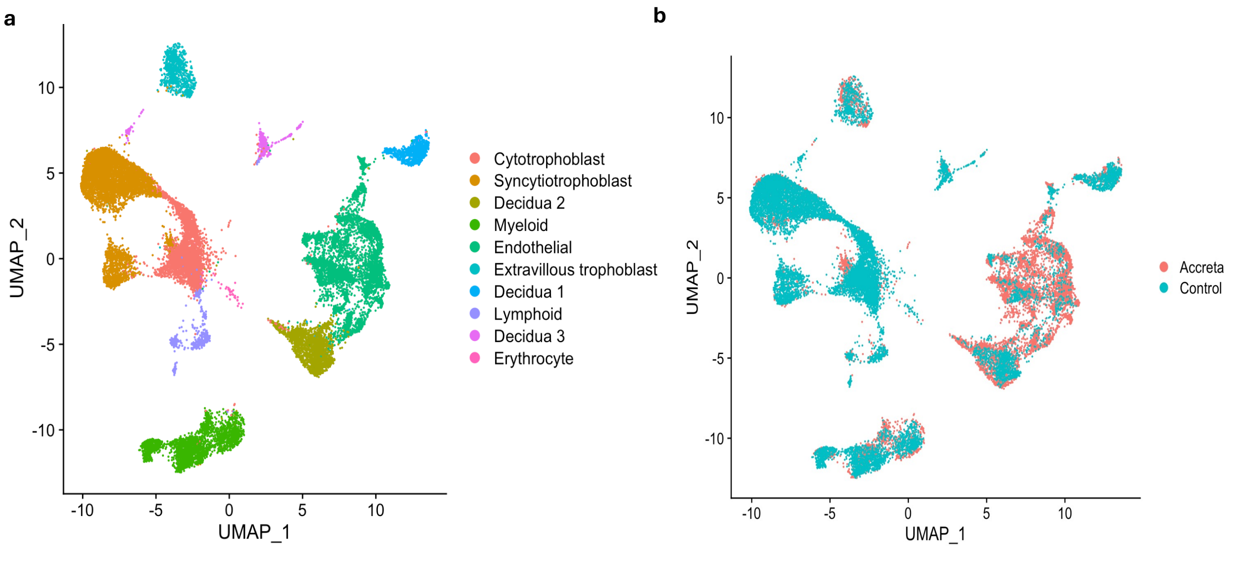

Supplementary Figure 5: Results of single-cell placenta accreta analysis.
a UMAP identifying cell type designation associated with gene clusters.
b UMAP representation split by disease phenotype of placenta accreta (n=3) and control (n=3).

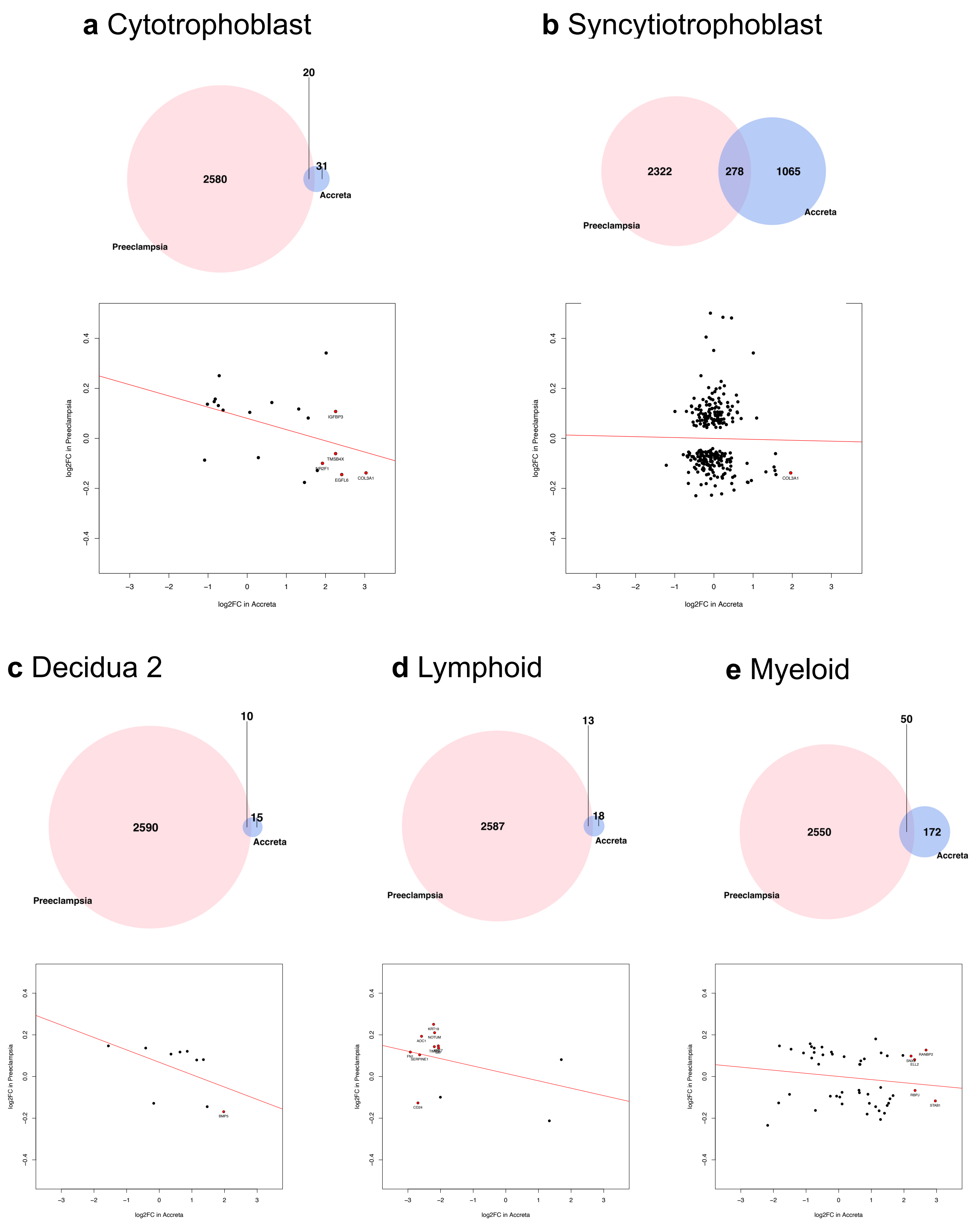

Supplementary Figure 6: Comparative transcriptomic analysis of early onset preeclampsia and placenta accreta, by cell type, additional cells with uncorrelated expression difference.

a Cytotrophoblast: Overlap of 20 genes. Significant genes with a log2FC of >2 between preeclampsia and accreta include *IGFBP3, TMSB4X, NR2F1, EGFL6, COL3A1* (red).
b Syncytiotrophoblast: Overlap of 278 genes. Significant genes with a log2FC of >2 between preeclampsia and accreta include *COL3A1* (red).
c Decidua 2: Overlap of 10 genes. Significant genes with a log2FC of >2 between preeclampsia and accreta include *BMP5* (red).
d Lymphoid: Overlap of 13 genes. Significant genes with a log2FC of >2 between preeclampsia and accreta include *KRT19, NOTUM, AOC1, FN1, SERPINE1, CD24, MIF, TIMP3, KRT7, GPX3* (red).
e Myeloid: Overlap of 50 genes. Significant genes with a log2FC of >2 between preeclampsia and accreta include *SNX9, ELL2, RANBP2, RBPJ, STAB1* (red).

Cell Type	Cell Number	Up #	Down #	Total Gene #
Cytotrophoblast	6531	32	19	51
Decidua1	1326	265	145	410
Decidua2	2435	20	5	25
Decidua3	913	782	1398	2180
Endothelial	6158	2023	1259	3282
Erythrocyte	602	0	0	0
Extravillous trophoblast	1637	780	933	1713
Lymphoid	1048	7	24	31
Myeloid	3963	137	85	222
Syncytiotrophoblast	6793	627	716	1343

Supplementary Table 1: Single-cell accreta analysis.
Number of cells and differentially expressed genes identified by cell based on disease phenotype for placenta accreta, indicating positive or negative expression compared to control.

Supplementary data sheets

metalimmapreeclampsiacontrol_preterm_preEonly_all.csv

Meta-analysis differential gene expression results for 4 preterm preeclampsia datasets (GSE25906, GSE74341, GSE75010, GSE93839) adjusted for cell type

metalimmaGSE136048.csv

Differential gene expression result for 1 placenta accreta dataset GSE136048

AccretaSingleCellDEG.xls

Placenta accreta single cell differential gene expression results- for each cell type, adjusted for batch and term.

metalimmaoverlap_2_120.csv

120 genes shared between preterm preeclampsia and placenta accreta arrays.

PreeclampsiaAccretaOverlapbyCell_BackgroundPreeclampsia.xls

Genes shared between preterm preeclampsia and single-cell placenta accreta analysis, by cell type, with expression values denoting the comparison between early onset preeclampsia and preterm controls.

datasetsearch.xlsx

Results of GEO dataset search and annotate of included and excluded datasets
